# Targeting tumor-necrosis factor receptor pathways for tumor immunotherapy

**DOI:** 10.1186/2051-1426-2-7

**Published:** 2014-04-15

**Authors:** David A Schaer, Daniel Hirschhorn-Cymerman, Jedd D Wolchok

**Affiliations:** 1 Swim Across America Laboratory, Immunology Program, Sloan-Kettering Institute for Cancer Research, New York, NY 10065, USA; 2 Weill Cornell Medical College, New York, NY 10065, USA; 3 Ludwig Collaborative Lab, New York, NY 10065, USA; 4 Ludwig Center for Cancer Immunotherapy at Memorial Sloan-Kettering Cancer Center, New York, NY 10065, USA; 5 Current address: Department of Cancer Immunobiology, ImClone Systems, a wholly-owned subsidiary of Eli Lilly & Co, New York, NY 10016, USA

**Keywords:** Cancer, Immunotherapy, 4-1BB, OX40, GITR

## Abstract

With the success of ipilimumab and promise of programmed death-1 pathway-targeted agents, the field of tumor immunotherapy is expanding rapidly. Newer targets for clinical development include select members of the tumor necrosis factor receptor (TNFR) family. Agonist antibodies to these co-stimulatory molecules target both T and B cells, modulating T-cell activation and enhancing immune responses. *In vitro* and *in vivo* preclinical data have provided the basis for continued development of 4-1BB, OX40, glucocorticoid-induced TNFR-related gene, herpes virus entry mediator, and CD27 as potential therapies for patients with cancer. In this review, we summarize the immune response to tumors, consider preclinical and early clinical data on select TNFR family members, discuss potential translational challenges and suggest possible combination therapies with the aim of inducing durable antitumor responses.

## Introduction

After nearly a century of skepticism regarding the efficacy of immunotherapy in cancer, a resurgence has begun that is driven primarily by the success of ipilimumab [1]. The immune-mediated mechanism of action and resultant antitumor activity of ipilimumab lend support to the notion that tumors are under immune surveillance. Moreover, the concept of “immuno-editing” suggests that the pressure exerted on tumors by the immune system shapes or “edits” tumor cells, allowing their escape from immune elimination [2]. Briefly, the immune system is capable of eliminating malignant cells during initial transformation. As tumors grow, an “equilibrium” is reached where immune tumor growth is matched by immune-mediated tumor destruction. Eventually, malignant cells either accumulate mutations, making them non-immunogenic, or immunosuppressive pathways become activated, allowing the tumor to escape immune recognition [2-5]. It now appears that targeting immunomodulatory mechanisms can tip the balance from escape back toward elimination. In addition to ipilimumab and other co-inhibitory checkpoints (i.e., programmed death-1 [PD-1]), research into the stimulation of T cell responses via agonist therapies has opened another therapeutic possibility. This review will focus on the rationale for targeting co-stimulatory pathways in T cells, summarize agents in development, and offer possible treatment strategies using these agents in combination with other immunotherapies.

## Review

The host response to tumors has been well described [6-9]. However, tumors have developed ways to escape this response via a number of mechanisms; these have been extensively reviewed previously [6-8,10]. The accumulation of suppressive cells and an inhibitory cytokine milieu in and around the tumor can form an immunosuppressive environment that prevents successful T cell-mediated destruction of malignant cells [11]. The goal of many immunotherapies is to help the immune system overcome the mechanisms that tumors employ to evade destruction.

### Targeting immune “checkpoint” pathways

Upon initial activation by antigen-presenting cells (APCs), tumor-specific T cells receive essential co-stimulation through binding of CD28 to CD80/86 on activated APCs. However, the CD28-CD80/86 pathway is antagonized by cytotoxic T lymphocyte antigen-4 (CTLA-4; CD152), one of the first co-inhibitory checkpoints that self-limit responses. CTLA-4 competes with CD28 for CD80/86 binding and has a much greater affinity than CD28 for CD80; CTLA-4 signaling decreases the magnitude of early T-cell activation, expansion, and function [12-14]. Ipilimumab blocks CTLA-4 antagonism of CD28 and was the first agent to demonstrate an overall survival benefit for patients with metastatic melanoma [1,15]. The ability of CTLA-4 blockade to enhance tumor immunity may be because most tumor antigens are modified self-antigens or mutated unique antigens against which T cell cytotoxic function is believed to be relatively poor. Therefore, by releasing this first brake on immune activation, ipilimumab allows the generation of a more effective immune response [16].

Subsequent to CTLA-4, PD-1 was also shown to regulate the immune response. PD-1 interacts with B7-homolog 1 (B7-H1 or programmed death receptor-1 ligand1 [PD-L1]) and B7-DC (PD-L2) on APCs and tumor cells. Ligation of PD-1 by either of its ligands downregulates T-cell receptor (TCR) signaling and abrogates stimulatory cytokine production [17,18]. Moreover, PD-1 upregulation on effector T cells identifies T cells with an exhausted phenotype that cannot maintain polyfunctional cytokine production [19]. Although the evaluation of PD-1/PD-L1–targeted therapies is at an early stage, they are showing promising results. Evaluation of the anti–PD-1 antibody nivolumab in more than 300 patients in a phase I trial, showed antitumor activity in patients with non-small cell lung cancer (NSCLC), renal cell carcinoma (RCC), and melanoma, and an encouraging safety profile [20,21]. Combination of nivolumab with ipilimumab has also demonstrated a potentially synergistic effect in a recent phase I combination study for patients with advanced melanoma [22]. Phase III trials in these tumors are ongoing. The anti–PD-1 antibody CT-011 has shown activity in a small phase I trial of patients with various hematological malignancies [23]. Data with other anti–PD-1 and anti–PD-L1 antibodies and fusion proteins are becoming available [24-27].

### TNFR superfamily costimulatory molecules

In addition to blocking co-inhibitory pathways, activating co-stimulatory pathways to potentiate antitumor immune responses is a promising approach. Members of the tumor necrosis factor receptor superfamily (TNFRsf) include several co-stimulatory proteins with key roles in B and T cell development, survival, immune activation, and antitumor immune responses [28]. These co-stimulatory TNFRsf members lack death domains, enabling them to enhance activation and proinflammatory cascades [29], making them attractive therapeutic targets (Table 1). Recent clinical success with checkpoint inhibitors has provided the rationale for investigating agonism of 4-1BB (CD137), OX40, glucocorticoid-induced TNFR-related gene (GITR), herpes virus entry mediator (HVEM), and CD27 in order to extend clinical benefit to more patients.

**Table 1:** TNFR-targeted agents in development

**TNF receptor molecule**	**Name**	**Description**	**Sponsor**	**Phase in cancer patients**
4-1BB (CD137)	CART19 cells	T cells transduced with antibody against CD19 linked to the intracellular signaling domains of 4-1BB and CD3-zeta	University of Pennsylvania and others	II
	Urelumab	Fully human anti-CD137 agonist monoclonal antibody	Bristol Myers-Squibb	I
OX40		Mouse monoclonal anti-OX40 agonist antibody	Providence Health & Services and others	II
		Humanized antibody against OX40		Preclinical
	hFcILZOX40L	Recombinant human Fc:OX40L fusion protein	Providence Portland Medical Center	Preclinical
GITR	TRX518	Humanized anti-GITR agonist monoclonal antibody	GITR, Inc.	I
	GITRL RNA DC	GITRL-expressing DCs	Duke University	I
CD27	CDX-1127	Fully human anti-CD27 agonist monoclonal antibody	Celldex Therapeutics	I

Abbreviation: *DCs* Dendritic Cells, *GITR* Glucocorticoid-Induced TNFR-Related Gene, *GITRL* GITR Ligand, *TNF* Tumor-Necrosis Factor, *TNFR* Tumor-Necrosis Factor Receptor.

#### 4-1BB (CD137)

4-1BB is a molecule with profound effects on T-cell proliferation and CD8^+^ T-cell function [30-32]. It is primarily present on activated but not resting T cells, activated natural killer (NK) cells, and natural killer T (NKT) cells. It is expressed constitutively on certain populations of dendritic cells (DCs) and regulatory T cells (Tregs) [33-36]; 4-1BB expression is upregulated on monocytes upon activation [35]. Stimulation of 4-1BB by either its natural ligand, 4-1BBL, or by agonist antibodies enhances the activation of various immune cells, including T cells, DC (upregulation of B7 molecules and immunostimulatory cytokine production), monocytes, and neutrophils (proinflammatory cytokine secretion), and induces a spectrum of effects on B and NK cells [35,36]. 4-1BB signaling can co-stimulate T cells in a CD28-independent manner [37], protect tumor-infiltrating lymphocytes (TILs) from activation-induced cell death (AICD) via upregulation of antiapoptotic pathways [38,39], and enhance cytotoxic T lymphocyte (CTL) survival and cytolytic activity [40].

Preclinical data show that targeting 4-1BB with an agonist antibody can promote tumor control in numerous preclinical models, and is often associated with increased CTL effector capability [36,41-43]. The effects of pairing agonist anti–4-1BB antibodies with other therapies have also been investigated. Anti-4-1BB antibody administered with adoptive T-cell therapy resulted in survival and resistance to rechallenge in 80% of mice bearing established thymomas [44]. Combining anti–4-1BB with an agonist anti-CD40 plus a blocking antibody against DR5, a receptor for TRAIL (a strategy termed “trimab”), had activity against multiple transplanted tumors in mice [45]. Adding anti–CTLA-4 further increased the potency of this approach, resulting in ~80% tumor rejection of established mammary 4 T1 tumors in mice [46]. Combining CTLA-4 blockade and 4-1BB co-stimulation with a granulocyte-macrophage colony-stimulating factor (GM-CSF)–secreting melanoma vaccine greatly improved tumor eradication and promoted survival compared with vaccine plus either agent [47]. Combination treatment increased proliferation and tumor infiltration by both CD4^+^ and CD8^+^ T cells, and intratumor inflammatory cytokine production. Likewise, a 4-1BBL–expressing RM-1 cell vaccine used with CTLA-4 blockade in mice bearing RM-1 prostate tumors improved survival compared with mice treated with monotherapy [48]. These data suggest that concurrent manipulation of the CTLA-4 and 4-1BB signaling pathways merits clinical evaluation.

Other combinations have also been investigated. Kohrt and colleagues showed human trastuzumab-activated NK cells had improved cytotoxicity against breast cancer cells when stimulated with an agonist 4-1BB antibody [49]. Agonistic anti–4-1BB antibodies also had antitumor activity when given together with radiation therapy in murine breast and lung cancer models and when combined with anti–PD-1 plus radiotherapy [50,51]. Finally, targeting 4-1BB with intratumoral interferon-α therapy or DC-based vaccination also produced significant antitumor responses and/or improved survival [52,53].

Against this background, agonist 4-1BB therapy with urelumab (BMS-663513) was investigated in patients with melanoma, RCC, and ovarian cancer. Although some antitumor activity was observed during a phase I trial, safety concerns halted development [54,55]. Development has recently restarted using urelumab at a lower dose in combination with other agents (NCT01471210 and NCT01775631, clincaltrials.gov).

A different approach to utilizing the immune-potentiating properties of 4-1BB has been explored in the context of chimeric antigen receptor (CAR)–transduced T cells. Expressing an antibody with specificity for the B cell antigen CD19 linked to the intracellular signaling domains of 4-1BB and CD3-ζ enhanced the survival and effector functions provided by transduced T cells directed against tumor cells. T cells with this modified CAR (termed “CART19” cells) infused into a patient with refractory chronic lymphocytic leukemia (CLL) induced complete disease remission that was ongoing for more than 10 months [56]. Clinical studies of CART19 cells in patients with various B cell leukemias and lymphomas are ongoing (clinicaltrials.gov).

#### OX40 (CD134)

OX40 (TNR4) [57] is similar to 4-1BB and GITR, and engagement promotes T-cell activation, survival, proliferation, and cytokine production [58-62]. OX40 is primarily expressed on activated CD4^+^ T cells 24–48 hours after activation, and on CD8^+^ T cells, neutrophils, dendritic cells, and Tregs [60,63]. The natural ligand of OX40, OX40 ligand (OX40L), is most common on APCs, and is also found on activated T cells. OX40 expression is induced by TCR stimulation, but co-stimulation through other molecules, such as CD28, or exposure to certain cytokines can further upregulate expression [64]. While OX40 has a key role in the development and function of Tregs, engagement of OX40 with an agonist antibody can also deactivate the suppressive function of Tregs [63,65-69].

OX40 agonism has been investigated in multiple tumor models. As a monotherapy, it delayed tumor growth *in vivo* and promoted the rejection of various tumors [60,62,70]. OX40-dependent antitumor immunity required both CD4^+^ and CD8^+^ T cells, and a significant proportion of treated mice remained tumor-free and resistant to rechallenge, supporting the notion that OX40 engagement promotes memory [71]. Combination approaches aimed at improving the efficacy of OX40 engagement have been explored. Combinations of anti-OX40 with fractionated radiotherapy, interleukin (IL)-12 and anti–4-1BB, anti–CTLA-4 and CpG oligonucleotides, anti-CD25 and anti–CTLA-4 with adoptive cell transfer, transforming growth factor (TGF)-β inhibition, or IL-2 improved antitumor responses, tumor rejection, long-term survival, and/or resistance to tumor rechallenge in mice bearing various cancers [64,71-75]. We have shown that combining anti-OX40 with cyclophosphamide led to the regression of the poorly immunogenic B16 murine melanoma and was associated with fewer intratumoral Tregs, leading to a favorable effector/Treg cell ratio within the tumor microenvironment [76]. Adoptive transfer of melanoma specific CD4^+^ T cells together with OX40 and cyclophosphamide eliminated even more advanced melanomas. This combination triggered cytolytic activity in the transferred CD4^+^ T cells, a phenomenon largely responsible for the potency of this combination [76].

In the first-in-human clinical trial, 30 patients with solid tumors received a murine anti-human OX40 monoclonal antibody at doses from 0.1 through 2 mg/kg; the maximum tolerated dose was not identified, and some patients had an objective response after one dose [77]. Humanized OX40 antibodies and a fully human OX40L:IgG fusion protein have been developed [60,78,79]. Phase II clinical trials evaluating OX40 agonists with stereotactic radiation and/or cyclophosphamide in patients with multiple tumor types are ongoing (NCT01642290, NCT01303705).

#### GITR (CD357)

GITR is expressed at low levels on resting CD4^+^ and CD8^+^ T cells, is upregulated after 24–72 hours of stimulation, and remains expressed for several days [80-82]. In contrast to effector T cells, Tregs constitutively express GITR. GITR has also been observed on DC, monocytes, and NK cells. Similar to OX40 and 4-1BB, ligation provides co-stimulatory signals that enhance T-cell proliferation and effector function, and protect T cells from AICD [81,82]. GITR ligand (GITRL) is highly expressed on activated APCs and endothelial cells (ECs). Interestingly, while the role of GITRL on APCs appears primarily to provide costimulation, it was recently shown that GITRL/GITR interactions on EC may be important for triggering leukocyte adhesion and transmigration [83]. Although human and mouse GITR have similar characteristics, unlike most TNFR family members, murine GITR exists as a dimer instead of a trimer [84,85]. How this difference relates to the biological functions of mouse and human GITR is unknown.

Reports showing GITR ligation can break self-tolerance and abrogate T-cell suppression by Tregs identify it as a target for cancer immunotherapy [86,87]. Our laboratory was the first to demonstrate that an agonist anti-GITR antibody, the rat monoclonal DTA-1, could protect mice from B16 tumor rechallenge and that treatment induced rejection of small, established B16 tumors [88,89]. Stimulation of GITR also cured established sarcomas, induced long-lasting memory, and had activity in other mouse models [82,90]. GITRL manipulation has also been explored. Mice bearing GITRL-expressing tumors had improved CTL effector function and peritumoral injection of a GITRL vaccine slowed the growth of established tumors [91].

Multiple mechanisms appear to contribute to the antitumor effects of GITR modulation. We have demonstrated that DTA-1 administration impaired intratumoral Treg expression of FoxP3, resulting in a loss of Treg lineage stability and abrogation of intratumor Treg suppressive function [92]. This led to a higher effector T cell (Teff):Treg ratio and improved antitumor CTL function [88]. Additionally, Côté and colleagues found that DTA-1 treatment enhanced the development of high-avidity CTL responses to tumor-associated antigens in a mouse melanoma model [93]. Recent reports show that DTA-1 can cause depletion of intratumoral Tregs through interactions between the antibody and activating FcγRs on myeloid cells [94]. Because of differences in tumor models and underlying treatment conditions, it is unclear if one of these mechanisms is dominant or if all play a role in GITR agonist immunotherapy. The exact contribution of each will require further investigation.

GITR agonism (either via DTA-1 or GITRL fusion protein) has been successfully combined with other immunotherapies, including DNA- or DC-based vaccines [93,95-97]. Response to DTA-1 in mice bearing fibrosarcoma or colorectal carcinoma improved in combination with adoptive T-cell transfer or an antagonistic anti–CTLA-4 antibody [90,98,99]. Pruitt and colleagues saw improvements in antitumor immunity when a melanoma DC vaccine was combined with DCs expressing anti–CTLA-4 and anti-GITR mRNA [100].

While most reports show GITR-GITRL interactions are co-stimulatory to T cells, data regarding the immune-stimulating potential of GITR on non–T-cell subsets, particularly NK cells, are conflicting. Human leukemia cell expression of GITR-L interferes with NK cell-mediated immunity through reverse signaling, which causes tumor production of suppressive cytokines, downregulation of co-stimulatory molecules, and evasion from immune detection [101,102]. Nonetheless, in collaboration with Ludwig Cancer Research, Cancer Research Institute, and GITR, Inc. (Cambridge, MA, USA), we initiated a phase I trial (NCT01239134) of the humanized anti-GITR antibody TRX518 [103]. A trial examining the effects of GITRL-expressing DCs (with or without DC expressing anti–CTLA-4) plus a DC tumor vaccine in patients with melanoma is also ongoing (NCT01216436).

#### HVEM (CD270)

HVEM, another member of the TNFR superfamily [104], has multiple binding partners and various downstream effects that can positively and negatively modulate T-cell activation [105]. HVEM expression kinetics are the opposite of 4-1BB, OX40, and GITR: HVEM is highly expressed on resting T cells but downregulated upon activation. When cells return to less-activated states, HVEM expression is restored. There is significant HVEM expression on naive and memory, but not activated, B cells. Other immune cells, including Tregs, NK cells, monocytes, and immature DCs, also express HVEM. There are at least four natural ligands for HVEM: the inhibitory Ig-related molecules CD160 and B and T lymphocyte attenuator (BTLA; capable of bidirectional signaling) and the stimulatory TNF-related cytokines LIGHT and lymphotoxin-alpha [106-109]. LIGHT is expressed upon T and B cell activation and on monocytes and immature DCs. BTLA is found on various lymphoid and myeloid cells, with particularly high expression on B cells and plasmacytoid DCs. CD160, by contrast, is observed only on subsets of T and NK cells [105]. While binding of LIGHT or lymphotoxin-α to HVEM triggers T and B cell stimulation and APC maturation and differentiation, CD160 or BTLA binding to HVEM on T or B cells has the reverse effect, inhibiting T and B cell activation.

Studies have explored the potential of the HVEM pathway as a therapeutic target. Murine P815 mastocytoma cells engineered to express the single-chain variable fragment (scFv) of an anti-HVEM agonistic monoclonal antibody stimulated proliferation and cytokine production in co-cultured T cells [110]. Expression of anti-HVEM scFv *in vivo* resulted in T-cell–dependent tumor rejection (in 80% of treated mice) and a lasting memory response. Combination of the anti-HVEM scFv-expressing tumor vaccine with anti–4-1BB monoclonal antibody therapy synergized to induce regression of more established tumors, leading to survival and memory responses not seen with either agent alone [111]. In melanoma patients, tumor-specific T cells were found to express BTLA concurrent with HVEM [108,109]. Fourcade and colleagues tested the significance of this expression, showing that BTLA blockade enhanced the proliferation and antitumor activity of melanoma-specific CD8^+^ T cells [109]. Approaches to mono- or combination therapy are presented in the review by Pasero and colleagues [105]. However, manipulating the HVEM signaling pathway(s) provides a significant challenge, requiring agents that precisely induce HVEM-related immunostimulatory effects or block HVEM-related immunosuppressive effects.

#### CD27

CD27 has a key role in the generation of immunological memory via effects on T-cell expansion and survival, and B cell development [112-114]. CD27 is constitutively expressed on conventional T cells (including populations of Tregs) and, like other members of the TNFRsf, is upregulated upon activation [115]. Expression of CD27 on human Tregs correlates with FoxP3 expression and suppressive functionality [116,117]. CD27 is also expressed on subsets of B cells, most strikingly on plasma cells, for which it is a broadly accepted marker [118]. CD70, the natural ligand for CD27, is transiently expressed on activated APCs and T cells [119]. Ligation of CD27 by CD70 has stimulatory effects on T-cell proliferation, expansion, and survival dependent upon IL-2 autocrine signaling [120].

Triggering CD27 signaling in CAR-transduced T cells can enhance effector function, survival, and improve antitumor activity in a xenograft mouse model of ovarian cancer [121]. Constitutive CD70 expression on tumors or DCs improved antitumor immunity in murine lymphoma models, enhancing NK-mediated rejection of class-1 deficient tumor cells via perforin- and IFN-γ-dependent mechanisms [122]. CD70 expression also leads to generation of memory T-cell response to secondary tumor challenge. Conversely, intact CD27/CD70 signaling has been associated with decreased antitumor immune responses and an increased intratumoral Tregs (potentially due to improved Treg resistance to apoptosis and increased IL-2 secretion by effector T cells). Compared with wild-type mice, tumors in CD27-deficient mice had decreased vascularization and slower growth [123]. Furthermore, wild-type mice treated with an anti-CD27 blocking antibody had fewer Tregs and slower tumor growth than untreated mice. In this setting, Tregs were critical mediators of CD27-dependent effects on tumor growth. Schurch and colleagues similarly demonstrated that CD27 triggering by CD70 ligation in chronic myelogenous leukemia stem cells accelerated disease progression and was associated with activation of the Wnt pathway [124]. Such data complicate the consideration of CD27 as a target for tumor immunotherapy. The effects of CD27 pathway triggering may depend upon the environment in which the stimulus is delivered—within the tumor microenvironment or in peripheral lymphoid tissues—as well as length of exposure to stimulating agents.

A fully human monoclonal CD27 agonist antibody, CDX-1127 (Celldex Therapeutics, Inc. Needham, MA, USA), is being evaluated in a phase I clinical trial in select hematological and solid tumors [125]. Preclinical experiments with a mouse homologue showed delayed human hematological tumor growth in xenograft mouse models and murine tumor growth in immunocompetent mice [126]. Again, similar to HVEM, continued clinical development of CD27-related agents will require teasing apart the multiple inhibitory and co-stimulatory mechanisms that target multiple cell populations, and precise triggering of certain molecules in select environments to avoid exaggerating tumor-induced immunosuppression. Since Tregs appear to be a critical target of CD27 agonism, perhaps prior Treg depletion may be appropriate.

## Conclusions

The success of cancer immunotherapy with agonist antibodies may vary according to tumor type, since the expression of the target molecules can differ across tumors (for example, the expression of HVEM in certain leukemias and lymphomas). Although a multifactorial approach to cancer therapy is attractive [127], the potential risk of autoimmune reactions and bystander tissue damage in the setting of immunostimulation should not be underestimated. The well-documented phase I study of the anti-CD28 monoclonal antibody TGN1412, in which six healthy volunteers suffered massive cytokine release and major organ damage, emphasized the need for extreme caution in trial design and execution when investigating immune activators [128]. It should be noted, however, that CD28 is constitutively expressed on T cells, whereas most of the TNFRsf members are upregulated upon activation. Therefore, targeting TNFRsf members might have more restricted downstream effects, including a more defined toxicity profile.

Targeting the co-stimulatory TNFRsf shows promise for the treatment of cancer. Although there have been life-threatening side effects with some agonist antibodies, there is obvious clinical activity, emphasizing the need for careful titration and dosing. From this perspective, it may not be necessary to “press on the gas” constantly if agonist antibodies are administered with a checkpoint blocker (i.e., anti-PD-1, anti-CTLA-4) to “remove the brake”.

The mechanisms of immune evasion are not the same for each patient’s tumor (even of the same tumor type) and as such, monotherapies may not achieve sufficient potency. Combinatorial approaches, which include targeting TNFRsf, show promise in preclinical studies. Thus, combination with chemotherapy or irradiation, which can induce immunogenic cell death and stimulate antitumor T cells, is a rational. Additionally, combining anti-TNFRsf antibodies with small molecule inhibitors should be considered. Although clinical studies of TNFRsf agonists are at an early stage, careful development may see additional therapies brought to patients who most need them most.

## Abbreviations

AICD: Activation-induced cell death; APCs: Antigen-presenting cells; B7-H1: B7-homolog 1; BTLA: B and T lymphocyte attenuator; CAR: Chimeric antigen receptor; CD137: 4-1BB; CLL: Chronic lymphocytic leukemia; CTL: Cytotoxic T lymphocyte; CTLA-4: Cytotoxic T lymphocyte antigen-4; DCs: Dendritic cells; ECs: Endothelial cells; GITR: Glucocorticoid-induced TNFR-related gene; GITRL: GITR ligand; GM-CSF: Granulocyte-macrophage colony-stimulating factor; HVEM: Herpes virus entry mediator; IL: Interleukin; MDSC: Myeloid-derived suppressor cells; NK: Natural killer; NKT: Natural killer T cells; NSCLC: Non-small cell lung cancer; OX40L: OX40 ligand; PD-1: Programmed death receptor-1; PD-L1/2: Programmed death receptor-1 ligand1/2; RCC: Renal cell carcinoma; scFv: Single-chain variable fragment; TCR: T-cell receptor; Teff: Effector T cell; TILs: Tumor-infiltrating lymphocytes; TNF: Tumor-necrosis factor; TNFR: Tumor-necrosis factor receptor; TNFRsf: Tumor necrosis factor receptor superfamily; Treg: Regulatory T cell.

## Competing interests

DS and DH declare no competing interest. JW discloses that he is a paid consultant for Medimmune and Bristol-Myers Squibb.

## Authors’ contributions

DS, DH and JW contributed equally to the development of this manuscript. All authors read and approved the final manuscript.

## References

[B1] Hodi FS O’Day SJ McDermott DF Weber RW Sosman JA Haanen JB Gonzalez R Robert C Schadendorf D Hassel JC Akerley W van den Eertwegh AJ Lutzky J Lorigan P Vaubel JM Linette GP Hogg D Ottensmeier CH Lebbé C Peschel C Quirt I Clark JI Wolchok JD Weber JS Tian J Yellin MJ Nichol GM Hoos A Urba WJ Improved survival with ipilimumab in patients with metastatic melanoma N Engl J Med 2010 363 711 723 10.1056/NEJMoa1003466 20525992 PMC3549297

[B2] DuPage M Mazumdar C Schmidt LM Cheung AF Jacks T Expression of tumour-specific antigens underlies cancer immunoediting Nature 2012 482 405 409 10.1038/nature10803 22318517 PMC3288744

[B3] Schreiber RD Old LJ Smyth MJ Cancer immunoediting: integrating immunity’s roles in cancer suppression and promotion Science 2011 331 1565 1570 10.1126/science.1203486 21436444

[B4] Dunn GP Bruce AT Ikeda H Old LJ Schreiber RD Cancer immunoediting: from immunosurveillance to tumor escape Nat Immunol 2002 3 991 998 10.1038/ni1102-991 12407406

[B5] Matsushita H Vesely MD Koboldt DC Rickert CG Uppaluri R Magrini VJ Arthur CD White JM Chen YS Shea LK Hundal J Wendl MC Demeter R Wylie T Allison JP Smyth MJ Old LJ Mardis ER Schreiber RD Cancer exome analysis reveals a T-cell-dependent mechanism of cancer immunoediting Nature 2012 482 400 404 10.1038/nature10755 22318521 PMC3874809

[B6] Draghiciu O Nijman HW Daemen T From tumor immunosuppression to eradication: targeting homing and activity of immune effector cells to tumors Clin Dev Immunol 2011 2011 439053 22190971 10.1155/2011/439053 PMC3235497

[B7] Mellman I Coukos G Dranoff G Cancer immunotherapy comes of age Nature 2011 480 480 489 10.1038/nature10673 22193102 PMC3967235

[B8] Töpfer K Kempe S Müller N Schmitz M Bachmann M Cartellieri M Schackert G Temme A Tumor evasion from T cell surveillance J Biomed Biotechnol 2011 2011 918471 22190859 10.1155/2011/918471 PMC3228689

[B9] Fuertes MB Kacha AK Kline J Woo SR Kranz DM Murphy KM Gajewski TF Host type I IFN signals are required for antitumor CD8+ T cell responses through CD8{alpha} + dendritic cells J Exp Med 2011 208 2005 2016 10.1084/jem.20101159 21930765 PMC3182064

[B10] Zou W Immunosuppressive networks in the tumour environment and their therapeutic relevance Nat Rev Cancer 2005 5 263 274 10.1038/nrc1586 15776005

[B11] Schaer DA Li Y Merghoub T Rizzuto GA Shemesh A Cohen AD Li Y Avogadri F Toledo-Crow R Houghton AN Wolchok JD Detection of intra-tumor self antigen recognition during melanoma tumor progression in mice using advanced multimode confocal/two photon microscope PLoS One 2011 6 e21214 10.1371/journal.pone.0021214 21731676 PMC3120835

[B12] Linsley PS Greene JL Brady W Bajorath J Ledbetter JA Peach R Human B7-1 (CD80) and B7-2 (CD86) bind with similar avidities but distinct kinetics to CD28 and CTLA-4 receptors Immunity 1994 1 793 801 10.1016/S1074-7613(94)80021-9 7534620

[B13] Teft WA Kirchhof MG Madrenas J A molecular perspective of CTLA-4 function Annu Rev Immunol 2006 24 65 97 10.1146/annurev.immunol.24.021605.090535 16551244

[B14] Krummel MF Allison JP CD28 and CTLA-4 have opposing effects on the response of T cells to stimulation J Exp Med 1995 182 459 465 10.1084/jem.182.2.459 7543139 PMC2192127

[B15] Korman A Yellin M Keler T Tumor immunotherapy: preclinical and clinical activity of anti-CTLA4 antibodies Curr Opin Investig Drugs 2005 6 582 591 15988909

[B16] Yuan J Adamow M Ginsberg BA Rasalan TS Ritter E Gallardo HF Xu Y Pogoriler E Terzulli SL Kuk D Panageas KS Ritter G Sznol M Halaban R Jungbluth AA Allison JP Old LJ Wolchok JD Gnjatic S Integrated NY-ESO-1 antibody and CD8+ T-cell responses correlate with clinical benefit in advanced melanoma patients treated with ipilimumab Proc Natl Acad Sci U S A 2011 108 16723 16728 10.1073/pnas.1110814108 21933959 PMC3189057

[B17] Freeman GJ Long AJ Iwai Y Bourque K Chernova T Nishimura H Fitz LJ Malenkovich N Okazaki T Byrne MC Horton HF Fouser L Carter L Ling V Bowman MR Carreno BM Collins M Wood CR Honjo T Engagement of the PD-1 immunoinhibitory receptor by a novel B7 family member leads to negative regulation of lymphocyte activation J Exp Med 2000 192 1027 1034 10.1084/jem.192.7.1027 11015443 PMC2193311

[B18] Chemnitz JM Parry RV Nichols KE June CH Riley JL SHP-1 and SHP-2 associate with immunoreceptor tyrosine-based switch motif of programmed death 1 upon primary human T cell stimulation, but only receptor ligation prevents T cell activation J Immunol 2004 173 945 954 10.4049/jimmunol.173.2.945 15240681

[B19] Barber DL Wherry EJ Masopust D Zhu B Allison JP Sharpe AH Freeman GJ Ahmed R Restoring function in exhausted CD8 T cells during chronic viral infection Nature 2006 439 682 687 10.1038/nature04444 16382236

[B20] Topalian SL Hodi FS Brahmer JR Gettinger SN Smith DC McDermott DF Powderly JD Carvajal RD Sosman JA Atkins MB Leming PD Spigel DR Antonia SJ Horn L Drake CG Pardoll DM Chen L Sharfman WH Anders RA Taube JM McMiller TL Xu H Korman AJ Jure-Kunkel M Agrawal S McDonald D Kollia GD Gupta A Wigginton JM Sznol M Safety, activity, and immune correlates of anti-PD-1 antibody in cancer N Engl J Med 2012 366 2443 2454 10.1056/NEJMoa1200690 22658127 PMC3544539

[B21] Topalian SL Brahmer JR Hodi FS McDermott DF Smith DC Gettinger S Taube JM Gupta A Wigginton JM Sznol M Anti-programmed death-1 (PD-1) (BMS-936558/MDX-1106/ONO-4538) in patients with advanced solid tumors: clinical activity, safety, and molecular markers [Abstract] Annals Oncol 2012 23 suppl 9 xi157

[B22] Wolchok JD Kluger H Callahan MK Postow MA Rizvi NA Lesokhin AM Segal NH Ariyan CE Gordon RA Reed K Burke MM Caldwell A Kronenberg SA Agunwamba BU Zhang X Lowy I Inzunza HD Feely W Horak CE Hong Q Korman AJ Wigginton JM Gupta A Sznol M Nivolumab plus ipilimumab in advanced melanoma N Engl J Med 2013 369 122 133 10.1056/NEJMoa1302369 23724867 PMC5698004

[B23] Berger R Rotem-Yehudar R Slama G Landes S Kneller A Leiba M Koren-Michowitz M Shimoni A Nagler A Phase I safety and pharmacokinetic study of CT-011, a humanized antibody interacting with PD-1, in patients with advanced hematologic malignancies Clin Cancer Res 2008 14 3044 3051 10.1158/1078-0432.CCR-07-4079 18483370

[B24] Patnaik A Kang SP Tolcher AW Rasco DW Papadopoulos KP Beeram M Drengler R Chen C Smith L Perez C Gergich K Lehnert M Phase I study of MK-3475 (anti-PD-1 monoclonal antibody) in patients with advanced solid tumors [Abstract] J Clin Oncol 2012 30 Suppl abstr 2512 10.1158/1078-0432.CCR-14-2607 25977344

[B25] MedImmune joins forces with leading cancer organizations to advance novel immunotherapy research [press release] https://www.medimmune.com/media/press-releases/2012/10/09/medimmune-joins-forces-with-leading-cancer-organizations-to-advance-novel-immunotherapy-research

[B26] Brahmer JR Tykodi SS Chow LQ Hwu WJ Topalian SL Hwu P Drake CG Camacho LH Kauh J Odunsi K Pitot HC Hamid O Bhatia S Martins R Eaton K Chen S Salay TM Alaparthy S Grosso JF Korman AJ Parker SM Agrawal S Goldberg SM Pardoll DM Gupta A Wigginton JM Safety and activity of anti-PD-L1 antibody in patients with advanced cancer N Engl J Med 2012 366 2455 2465 10.1056/NEJMoa1200694 22658128 PMC3563263

[B27] GlaxoSmithKline and Amplimmune form global strategic collaboration [press release] http://www.prnewswire.com/news-releases/glaxosmithkline-and-amplimmune-form-global-strategic-collaboration-99938599.html

[B28] Watts TH TNF/TNFR family members in costimulation of T cell responses Annu Rev Immunol 2005 23 23 68 10.1146/annurev.immunol.23.021704.115839 15771565

[B29] So T Lee SW Croft M Tumor necrosis factor/tumor necrosis factor receptor family members that positively regulate immunity Int J Hematol 2006 83 1 11 10.1532/IJH97.05120 16443545

[B30] Kwon BS Weissman SM cDNA sequences of two inducible T-cell genes Proc Natl Acad Sci U S A 1989 86 1963 1967 10.1073/pnas.86.6.1963 2784565 PMC286825

[B31] Pollok KE Kim YJ Zhou Z Hurtado J Kim KK Pickard RT Kwon BS Inducible T cell antigen 4-1BB. Analysis of expression and function J Immunol 1993 150 771 781 7678621

[B32] Shuford WW Klussman K Tritchler DD Loo DT Chalupny J Siadak AW Brown TJ Emswiler J Raecho H Larsen CP Pearson TC Ledbetter JA Aruffo A Mittler RS 4-1BB costimulatory signals preferentially induce CD8+ T cell proliferation and lead to the amplification *in vivo* of cytotoxic T cell responses J Exp Med 1997 186 47 55 10.1084/jem.186.1.47 9206996 PMC2198949

[B33] Wilcox RA Chapoval AI Gorski KS Otsuji M Shin T Flies DB Tamada K Mittler RS Tsuchiya H Pardoll DM Chen L Cutting edge: expression of functional CD137 receptor by dendritic cells J Immunol 2002 168 4262 4267 10.4049/jimmunol.168.9.4262 11970964

[B34] McHugh RS Whitters MJ Piccirillo CA Young DA Shevach EM Collins M Byrne MC CD4(+)CD25(+) immunoregulatory T cells: gene expression analysis reveals a functional role for the glucocorticoid-induced TNF receptor Immunity 2002 16 311 323 10.1016/S1074-7613(02)00280-7 11869690

[B35] Vinay DS Kwon BS 4-1BB signaling beyond T cells Cell Mol Immunol 2011 8 281 284 10.1038/cmi.2010.82 21217771 PMC4002439

[B36] Vinay DS Kwon BS Immunotherapy of cancer with 4-1BB Mol Cancer Ther 2012 11 1062 1070 10.1158/1535-7163.MCT-11-0677 22532596

[B37] Saoulli K Lee SY Cannons JL Yeh WC Santana A Goldstein MD Bangia N DeBenedette MA Mak TW Choi Y Watts TH CD28-independent, TRAF2-dependent costimulation of resting T cells by 4–1BB ligand J Exp Med 1998 187 1849 1862 10.1084/jem.187.11.1849 9607925 PMC2212301

[B38] Lee HW Park SJ Choi BK Kim HH Nam KO Kwon BS 4-1BB promotes the survival of CD8+ T lymphocytes by increasing expression of Bcl-xL and Bfl-1 J Immunol 2002 169 4882 4888 10.4049/jimmunol.169.9.4882 12391199

[B39] Stärck L Scholz C Dörken B Daniel PT Costimulation by CD137/4-1BB inhibits T cell apoptosis and induces Bcl-xL and c-FLIP(short) via phosphatidylinositol 3-kinase and AKT/protein kinase B Eur J Immunol 2005 35 1257 1266 10.1002/eji.200425686 15761847

[B40] Hernandez-Chacon JA Li Y Wu RC Bernatchez C Wang Y Weber J Hwu P Radvanyi LG Costimulation through the CD137/4-1BB pathway protects human melanoma tumor-infiltrating lymphocytes from activation-induced cell death and enhances antitumor effector function J Immunother 2011 34 236 250 10.1097/CJI.0b013e318209e7ec 21389874 PMC3063939

[B41] Melero I Shuford WW Newby SA Aruffo A Ledbetter JA Hellström KE Mittler RS Chen L Monoclonal antibodies against the 4-1BB T-cell activation molecule eradicate established tumors Nat Med 1997 3 682 685 10.1038/nm0697-682 9176498

[B42] Sabel MS Conway TF Chen FA Bankert RB Monoclonal antibodies directed against the T-cell activation molecule CD137 (interleukin-A or4-1BB) block human lymphocyte-mediated suppression of tumor xenografts in severe combined immunodeficient mice J Immunother 2000 23 362 368 10.1097/00002371-200005000-00009 10838665

[B43] Kim JA Averbook BJ Chambers K Rothchild K Kjaergaard J Papay R Shu S Divergent effects of 4-1BB antibodies on antitumor immunity and on tumor-reactive T-cell generation Cancer Res 2001 61 2031 2037 11280763

[B44] Lin GH Liu Y Ambagala T Kwon BS Ohashi PS Watts TH Evaluating the cellular targets of anti-4-1BB agonist antibody during immunotherapy of a pre-established tumor in mice PLoS One 2010 5 e11003 10.1371/journal.pone.0011003 20543982 PMC2882368

[B45] Uno T Takeda K Kojima Y Yoshizawa H Akiba H Mittler RS Gejyo F Okumura K Yagita H Smyth MJ Eradication of established tumors in mice by a combination antibody-based therapy Nat Med 2006 12 693 698 10.1038/nm1405 16680149

[B46] Takeda K Kojima Y Uno T Hayakawa Y Teng MW Yoshizawa H Yagita H Gejyo F Okumura K Smyth MJ Combination therapy of established tumors by antibodies targeting immune activating and suppressing molecules J Immunol 2010 184 5493 5501 10.4049/jimmunol.0903033 20400706

[B47] Curran MA Kim M Montalvo W Al-Shamkhani A Allison JP Combination CTLA-4 blockade and 4-1BB activation enhances tumor rejection by increasing T-cell infiltration, proliferation, and cytokine production PLoS One 2011 6 e19499 10.1371/journal.pone.0019499 21559358 PMC3085474

[B48] Youlin K Li Z Xiaodong W Xiuheng L Hengchen Z Combination immunotherapy with 4–1 BBL and CTLA-4 blockade for the treatment of prostate cancer Clin Dev Immunol 2012 2012 439235 22312406 10.1155/2012/439235 PMC3270651

[B49] Kohrt HE Houot R Weiskopf K Goldstein MJ Scheeren F Czerwinski D Colevas AD Weng WK Clarke MF Carlson RW Stockdale FE Mollick JA Chen L Levy R Stimulation of natural killer cells with a CD137-specific antibody enhances trastuzumab efficacy in xenotransplant models of breast cancer J Clin Invest 2012 122 1066 1075 10.1172/JCI61226 22326955 PMC3287235

[B50] Shi W Siemann DW Augmented antitumor effects of radiation therapy by 4-1BB antibody (BMS-469492) treatment Anticancer Res 2006 26 3445 3453 17094465

[B51] Verbrugge I Hagekyriakou J Sharp LL Galli M West A McLaughlin NM Duret H Yagita H Johnstone RW Smyth MJ Haynes NM Radiotherapy increases the permissiveness of established mammary tumors to rejection by immunomodulatory antibodies Cancer Res 2012 72 3163 3174 10.1158/0008-5472.CAN-12-0210 22570253

[B52] Dubrot J Palazón A Alfaro C Azpilikueta A Ochoa MC Rouzaut A Martinez-Forero I Teijeira A Berraondo P Le Bon A Hervás-Stubbs S Melero I Intratumoral injection of interferon-α and systemic delivery of agonist anti-CD137 monoclonal antibodies synergize for immunotherapy Int J Cancer 2011 128 105 118 10.1002/ijc.25333 20309938

[B53] Lee H Park HJ Sohn HJ Kim JM Kim SJ Combinatorial therapy for liver metastatic colon cancer: dendritic cell vaccine and low-dose agonistic anti-4-1BB antibody co-stimulatory signal J Surg Res 2011 169 e43 e50 10.1016/j.jss.2011.03.067 21571303

[B54] Ascierto PA Simeone E Sznol M Fu YX Melero I Clinical experiences with anti-CD137 and anti-PD1 therapeutic antibodies Semin Oncol 2010 37 508 516 10.1053/j.seminoncol.2010.09.008 21074066

[B55] Hwu W-J Targeted therapy for metastatic melanoma: from bench to bedside http://www.healio.com/hematology-oncology/melanoma-skin-cancer/news/print/hematology-oncology/%7B77E71A11-1FD1-4193-A2F7-7C50C6121C1F%7D/Targeted-therapy-for-metastatic-melanoma-From-bench-to-bedside

[B56] Porter DL Levine BL Kalos M Bagg A June CH Chimeric antigen receptor-modified T cells in chronic lymphoid leukemia N Engl J Med 2011 365 725 733 10.1056/NEJMoa1103849 21830940 PMC3387277

[B57] Paterson DJ Jefferies WA Green JR Brandon MR Corthesy P Puklavec M Williams AF Antigens of activated rat T lymphocytes including a molecule of 50,000 Mr detected only on CD4 positive T blasts Mol Immunol 1987 24 1281 1290 10.1016/0161-5890(87)90122-2 2828930

[B58] Baum PR Gayle RB 3rd Ramsdell F Srinivasan S Sorensen RA Watson ML Seldin MF Baker E Sutherland GR Clifford KN Alderson MR Goodwin RG Fanslow WC Molecular characterization of murine and human OX40/OX40 ligand systems: identification of a human OX40 ligand as the HTLV-1-regulated protein gp34 EMBO J 1994 13 3992 4001 8076595 10.1002/j.1460-2075.1994.tb06715.x PMC395319

[B59] Godfrey WR Fagnoni FF Harara MA Buck D Engleman EG Identification of a human OX-40 ligand, a costimulator of CD4+ T cells with homology to tumor necrosis factor J Exp Med 1994 180 757 762 10.1084/jem.180.2.757 7913952 PMC2191595

[B60] Weinberg AD Morris NP Kovacsovics-Bankowski M Urba WJ Curti BD Science gone translational: the OX40 agonist story Immunol Rev 2011 244 218 231 10.1111/j.1600-065X.2011.01069.x 22017441 PMC3622727

[B61] Pardee AD McCurry D Alber S Hu P Epstein AL Storkus WJ A therapeutic OX40 agonist dynamically alters dendritic, endothelial, and T cell subsets within the established tumor microenvironment Cancer Res 2010 70 9041 9052 10.1158/0008-5472.CAN-10-1369 21045144 PMC3058673

[B62] Jensen SM Maston LD Gough MJ Ruby CE Redmond WL Crittenden M Li Y Puri S Poehlein CH Morris N Kovacsovics-Bankowski M Moudgil T Twitty C Walker EB Hu HM Urba WJ Weinberg AD Curti BD Fox BA Signaling through OX40 enhances antitumor immunity Semin Oncol 2010 37 524 532 10.1053/j.seminoncol.2010.09.013 21074068 PMC2997672

[B63] Takeda I Ine S Killeen N Ndhlovu LC Murata K Satomi S Sugamura K Ishii N Distinct roles for theOX40-40 ligand interaction in regulatory and nonregulatory T cells J Immunol 2004 172 3580 3589 10.4049/jimmunol.172.6.3580 15004159

[B64] Redmond WL Triplett T Floyd K Weinberg AD Dual anti-OX40/IL-2 therapy augments tumor immunotherapy via IL-2R-mediated regulation of OX40 expression PLoS One 2012 7 e34467 10.1371/journal.pone.0034467 22496812 PMC3319580

[B65] Griseri T Asquith M Thompson C Powrie F OX40 is required for regulatory T cell-mediated control of colitis J Exp Med 2010 207 699 709 10.1084/jem.20091618 20368580 PMC2856021

[B66] Piconese S Pittoni P Burocchi A Gorzanelli A Carè A Tripodo C Colombo MP A non-redundant role for OX40 in the competitive fitness of Treg in response to IL-2 Eur J Immunol 2010 40 2902 2913 10.1002/eji.201040505 20806292

[B67] Ruby CE Yates MA Hirschhorn-Cymerman D Chlebeck P Wolchok JD Houghton AN Offner H Weinberg AD Cutting edge: OX40agonists can drive regulatory T cell expansion if the cytokine milieu is right J Immunol 2009 183 4853 4857 10.4049/jimmunol.0901112 19786544 PMC4625917

[B68] Valzasina B Guiducci C Dislich H Killeen N Weinberg AD Colombo MP Triggering of OX40 (CD134) on CD4 + CD25+ T cells blocks their inhibitory activity: a novel regulatory role for OX40 and its comparison with GITR Blood 2005 105 2845 2851 10.1182/blood-2004-07-2959 15591118

[B69] Croft M Control of immunity by the TNFR*-*related molecule OX40 (CD134) Annu Rev Immunol 2010 28 57 78 10.1146/annurev-immunol-030409-101243 20307208 PMC2882161

[B70] Weinberg AD Rivera MM Prell R Morris A Ramstad T Vetto JT Urba WJ Alvord G Bunce C Shields J Engagement of the OX-40 receptor *in vivo* enhances antitumor immunity J Immunol 2000 164 2160 2169 10.4049/jimmunol.164.4.2160 10657670

[B71] Gough MJ Crittenden MR Sarff MC Pang P Seung SK Vetto JT Hu HM Redmond WL Holland J Weinberg AD Adjuvant therapy with agonistic antibodies to CD134 (OX40) increases local control after surgical or radiation therapy of cancer in mice J Immunother 2010 33 798 809 10.1097/CJI.0b013e3181ee7095 20842057 PMC3563298

[B72] Pan PY Zang Y Weber K Meseck ML Chen SH OX40 ligation enhances primary and memory cytotoxic T lymphocyte responses in an immunotherapy for hepatic colon metastases Mol Ther 2002 6 528 536 10.1006/mthe.2002.0699 12377195

[B73] Houot R Levy R T-cell modulation combined with intratumoral CpG cures lymphoma in a mouse model without the need for chemotherapy Blood 2009 113 3546 3552 10.1182/blood-2008-07-170274 18941113 PMC2668854

[B74] Watanabe A Hara M Chosa E Nakamura K Sekiya R Shimizu T Onitsuka T Combination of adoptive cell transfer and antibody injection can eradicate established tumors in mice–an *in vivo* study using anti-OX40mAb, anti-CD25mAb and anti-CTLA4mAb Immunopharmacol Immunotoxicol 2010 32 238 245 10.3109/08923970903222355 20001272

[B75] Garrison K Hahn T Lee WC Ling LE Weinberg AD Akporiaye ET The small molecule TGF-β signaling inhibitor SM16 synergizes with agonistic OX40 antibody to suppress established mammary tumors and reduce spontaneous metastasis Cancer Immunol Immunother 2012 61 511 521 10.1007/s00262-011-1119-y 21971588 PMC3595193

[B76] Hirschhorn-Cymerman D Rizzuto GA Merghoub T Cohen AD Avogadri F Lesokhin AM Weinberg AD Wolchok JD Houghton AN OX40 engagement and chemotherapy combination provides potent antitumor immunity with concomitant regulatory T cell apoptosis J Exp Med 2009 206 1103 1116 10.1084/jem.20082205 19414558 PMC2715041

[B77] Curti BD Kovacsovics-Bankowski M Morris N Walker E Chisholm L Floyd K Walker J Gonzalez I Meeuwsen T Fox BA Moudgil T Miller W Haley D Coffey T Fisher B Delanty-Miller L Rymarchyk N Kelly T Crocenzi T Bernstein E Sanborn R Urba WJ Weinberg AD OX40 is a potent immune-stimulating target in late-stage cancer patients Cancer Res 2013 73 7189 98 10.1158/0008-5472.CAN-12-4174 24177180 PMC3922072

[B78] Voo KS Bover L Harline ML Vien LT Facchinetti V Arima K Kwak LW Liu YJ Antibodies targeting human OX40 expand effector T cells and block inducible and natural regulatory T cell function J Immunol 2013 191 3641 3650 10.4049/jimmunol.1202752 24014877 PMC3812678

[B79] Morris NP Peters C Montler R Hu HM Curti BD Urba WJ Weinberg AD Development and characterization of recombinant human Fc:OX40L fusion protein linked via a coiled-coil trimerization domain Mol Immunol 2007 44 3112 3121 10.1016/j.molimm.2007.02.004 17374396 PMC1950941

[B80] Nocentini G Giunchi L Ronchetti S Krausz LT Bartoli A Moraca R Migliorati G Riccardi C A new member of the tumor necrosis factor/nerve growth factor receptor family inhibits T cell receptor-induced apoptosis Proc Natl Acad Sci U S A 1997 94 6216 6221 10.1073/pnas.94.12.6216 9177197 PMC21029

[B81] Nocentini G Ronchetti S Petrillo MG Riccardi C Pharmacological modulation of GITRL/GITR system: therapeutic perspectives Br J Pharmacol 2012 165 2089 2099 10.1111/j.1476-5381.2011.01753.x 22029729 PMC3413846

[B82] Schaer DA Murphy JT Wolchok JD Modulation of GITR for cancer immunotherapy Curr Opin Immunol 2012 24 217 224 10.1016/j.coi.2011.12.011 22245556 PMC3413251

[B83] Lacal PM Petrillo MG Ruffini F Muzi A Bianchini R Ronchetti S Migliorati G Riccardi C Graziani G Nocentini G Glucocorticoid-induced tumor necrosis factor receptor family-related ligand triggering upregulates vascular cell adhesion molecule-1 and intercellular adhesion molecule-1 and promotes leukocyte adhesion J Pharmacol Exp Ther 2013 347 164 172 10.1124/jpet.113.207605 23892569

[B84] Chattopadhyay K Ramagopal UA Brenowitz M Nathenson SG Almo SC Evolution of GITRL immune function: murine GITRL exhibits unique structural and biochemical properties within the TNF superfamily Proc Natl Acad Sci U S A 2008 105 635 640 10.1073/pnas.0710529105 18182486 PMC2206588

[B85] Zhou Z Tone Y Song X Furuuchi K Lear JD Waldmann H Tone M Greene MI Murali R Structural basis for ligand-mediated mouse GITR activation Proc Natl Acad Sci USA 2008 105 641 645 10.1073/pnas.0711206105 18178614 PMC2206589

[B86] Shimizu J Yamazaki S Takahashi T Ishida Y Sakaguchi S Stimulation of CD25(+)CD4(+) regulatory T cells through GITR breaks immunological self-tolerance Nat Immunol 2002 3 135 142 10.1038/ni759 11812990

[B87] Stephens GL McHugh RS Whitters MJ Young DA Luxenberg D Carreno BM Collins M Shevach EM Engagement of glucocorticoid-induced TNFR family-related receptor on effector T cells by its ligand mediates resistance to suppression by CD4 + CD25+ T cells J Immunol 2004 173 5008 5020 10.4049/jimmunol.173.8.5008 15470044

[B88] Cohen AD Schaer DA Liu C Li Y Hirschhorn-Cymmerman D Kim SC Diab A Rizzuto G Duan F Perales MA Merghoub T Houghton AN Wolchok JD Agonist anti-GITR monoclonal antibody induces melanoma tumor immunity in mice by altering regulatory T cell stability and intra-tumor accumulation PLoS One 2010 5 e10436 10.1371/journal.pone.0010436 20454651 PMC2862699

[B89] Turk MJ Guevara-Patiño JA Rizzuto GA Engelhorn ME Sakaguchi S Houghton AN Concomitant tumor immunity to a poorly immunogenic melanoma is prevented by regulatory T cells J Exp Med 2004 200 771 782 10.1084/jem.20041130 15381730 PMC2211964

[B90] Ko K Yamazaki S Nakamura K Nishioka T Hirota K Yamaguchi T Shimizu J Nomura T Chiba T Sakaguchi S Treatment of advanced tumors with agonistic anti-GITR mAb and its effects on tumor-infiltrating Foxp3 + CD25 + CD4+ regulatory T cells J Exp Med 2005 202 885 891 10.1084/jem.20050940 16186187 PMC2213162

[B91] Piao J Kamimura Y Iwai H Cao Y Kikuchi K Hashiguchi M Masunaga T Jiang H Tamura K Sakaguchi S Azuma M Enhancement of T-cell-mediated anti-tumour immunity via the ectopically expressed glucocorticoid-induced tumour necrosis factor receptor-related receptor ligand (GITRL) on tumours Immunology 2009 127 489 499 10.1111/j.1365-2567.2008.03036.x 19604302 PMC2729526

[B92] Schaer DA Budhu S Liu C Bryson CF Malandro NM Cohen AD Zhong H Yang X Houghton AN Merghoub T Wolchok JD GITR pathway activation abrogates tumor immune suppression through loss of regulatory T cell lineage stability Cancer Immunol Res 2013 1 320 331 10.1158/2326-6066.CIR-13-0086 24416730 PMC3885345

[B93] Cohen AD Diab A Perales MA Wolchok JD Rizzuto G Merghoub T Huggins D Liu C Turk MJ Restifo NP Sakaguchi S Houghton AN Agonist anti-GITR antibody enhances vaccine-induced CD8(+) T-cell responses and tumor immunity Cancer Res 2006 66 4904 4912 10.1158/0008-5472.CAN-05-2813 16651447 PMC2242844

[B94] Bulliard Y Jolicoeur R Windman M Rue SM Ettenberg S Knee DA Wilson NS Dranoff G Brogdon JL Activating Fc γ receptors contribute to the antitumor activities of immunoregulatory receptor-targeting antibodies J Exp Med 2013 210 1685 1693 10.1084/jem.20130573 23897982 PMC3754864

[B95] Nishikawa H Kato T Hirayama M Orito Y Sato E Harada N Gnjatic S Old LJ Shiku H Regulatory T cell-resistant CD8+ T cells induced by glucocorticoid-induced tumor necrosisfactor receptor signaling Cancer Res 2008 68 5948 5954 10.1158/0008-5472.CAN-07-5839 18632650

[B96] Boczkowski D Lee J Pruitt S Nair S Dendritic cells engineered to secrete anti-GITR antibodies are effective adjuvants to dendritic cell-based immunotherapy Cancer Gene Ther 2009 16 900 911 10.1038/cgt.2009.39 19498460

[B97] Hoffmann C Stanke J Kaufmann AM Loddenkemper C Schneider A Cichon G Combining T-cell vaccination and application of agonistic anti-GITR mAb (DTA-1) induces complete eradication of HPV oncogene expressing tumors in mice J Immunother 2010 33 136 145 10.1097/CJI.0b013e3181badc46 20145549

[B98] Imai N Ikeda H Tawara I Wang L Wang L Nishikawa H Kato T Shiku H Glucocorticoid-induced tumor necrosis factor receptor stimulation enhances the multifunctionality of adoptively transferred tumor antigen-specific CD8+ T cells with tumor regression Cancer Sci 2009 100 1317 1325 10.1111/j.1349-7006.2009.01179.x 19432889 PMC11158648

[B99] Mitsui J Nishikawa H Muraoka D Wang L Noguchi T Sato E Kondo S Allison JP Sakaguchi S Old LJ Kato T Shiku H Two distinct mechanisms of augmented antitumor activity by modulation of immunostimulatory/inhibitory signals Clin Cancer Res 2010 16 2781 2791 10.1158/1078-0432.CCR-09-3243 20460483

[B100] Pruitt SK Boczkowski D de Rosa N Haley NR Morse MA Tyler DS Dannull J Nair S Enhancement of anti-tumor immunity through local modulation of CTLA-4 and GITR by dendritic cells Eur J Immunol 2011 41 3553 3563 10.1002/eji.201141383 22028176 PMC3594439

[B101] Baessler T Krusch M Schmiedel BJ Kloss M Baltz KM Wacker A Schmetzer HM Salih HR Glucocorticoid-induced tumor necrosis factor receptor-related protein ligand subverts immunosurveillance of acute myeloid leukemia in humans Cancer Res 2009 69 1037 1045 19155305 10.1158/0008-5472.CAN-08-2650

[B102] Baltz KM Krusch M Bringmann A Brossart P Mayer F Kloss M Baessler T Kumbier I Peterfi A Kupka S Kroeber S Menzel D Radsak MP Rammensee HG Salih HR Cancer immunoediting by GITR (glucocorticoid-induced TNF-related protein) ligand in humans: NK cell/tumor cell interactions FASEB J 2007 21 2442 2454 10.1096/fj.06-7724com 17360848

[B103] Rosenzweig M Ponte J Apostolou I Doty D Guild G Slavonic M Ponath P Vaickus L Development of TRX518, an aglycosyl humanized monoclonal antibody (Mab) agonist of huGITR [abstract] J Clin Oncol 2010 28 e13028

[B104] Kwon BS Tan KB Ni J Oh KO Lee ZH Kim KK Kim YJ Wang S Gentz R Yu GL Harrop J Lyn SD Silverman C Porter TG Truneh A Young PR A newly identified member of the tumor necrosis factor receptor superfamily with a wide tissue distribution and involvement in lymphocyte activation J Biol Chem 1997 272 14272 14276 10.1074/jbc.272.22.14272 9162061

[B105] Pasero C Speiser DE Derré L Olive D The HVEM network: new directions in targeting novel costimulatory/co-inhibitory molecules for cancer therapy Curr Opin Pharmacol 2012 12 475 485 10.1016/j.coph.2012.03.001 22445654

[B106] Morel Y Truneh A Sweet RW Olive D Costello RT The TNF superfamily members LIGHT and CD154 (CD40 ligand) costimulate induction of dendritic cell maturation and elicit specific CTL activity J Immunol 2001 167 2479 2486 10.4049/jimmunol.167.5.2479 11509586

[B107] Cai G Freeman GJ The CD160, BTLA, LIGHT/HVEM pathway: a bidirectional switch regulating T-cell activation Immunol Rev 2009 229 244 258 107 10.1111/j.1600-065X.2009.00783.x 19426226

[B108] Derré L Rivals JP Jandus C Pastor S Rimoldi D Romero P Michielin O Olive D Speiser DE BTLA mediates inhibition of human tumor-specific CD8+ T cells that can be partially reversed by vaccination J Clin Invest 2010 120 157 167 10.1172/JCI40070 20038811 PMC2799219

[B109] Fourcade J Sun Z Pagliano O Guillaume P Luescher IF Sander C Kirkwood JM Olive D Kuchroo V Zarour HM CD8+ T cells specific for tumor antigens can be rendered dysfunctional by the tumor microenvironment through upregulation of the inhibitory receptors BTLA and PD-1 Cancer Res 2012 72 887 896 10.1158/0008-5472.CAN-11-2637 22205715 PMC3288235

[B110] Cheung KJ Johnson NA Affleck JG Severson T Steidl C Ben- Neriah S Schein J Morin RD Moore R Shah SP Qian H Paul JE Telenius A Relander T Lam W Savage K Connors JM Brown C Marra MA Gascoyne RD Horsman DE Acquired TNFRSF14 mutations in follicular lymphoma are associated with worse prognosis Cancer Res 2010 70 9166 9174 10.1158/0008-5472.CAN-10-2460 20884631

[B111] Park JJ Anand S Zhao Y Matsumura Y Sakoda Y Kuramasu A Strome SE Chen L Tamada K Expression of anti-HVEM single-chain antibody on tumor cells induces tumor-specific immunity with long-term memory Cancer Immunol Immunother 2012 61 203 214 10.1007/s00262-011-1101-8 21877247 PMC3746333

[B112] van Lier RA Borst J Vroom TM Klein H Van Mourik P Zeijlemaker WP Melief CJ Tissue distribution and biochemical and functional properties of Tp55 (CD27), a novel T cell differentiation antigen J Immunol 1987 139 1589 1596 2442250

[B113] Hendriks J Gravestein LA Tesselaar K van Lier RA Schumacher TN Borst J CD27 is required for generation and long-term maintenance of T cell immunity Nat Immunol 2000 1 433 440 10.1038/80877 11062504

[B114] Denoeud J Moser M Role of CD27/CD70 pathway of activation in immunity and tolerance J Leukoc Biol 2011 89 195 203 10.1189/jlb.0610351 20699361

[B115] Croft M The role of TNF superfamily members in T-cell function and diseases Nat Rev Immunol 2009 9 271 285 10.1038/nri2526 19319144 PMC2737409

[B116] Ruprecht CR Gattorno M Ferlito F Gregorio A Martini A Lanzavecchia A Sallusto F Coexpression of CD25 and CD27 identifies FoxP3+ regulatory T cells in inflamed synovia J Exp Med 2005 201 1793 1803 10.1084/jem.20050085 15939793 PMC2213274

[B117] Coenen JJ Koenen HJ van Rijssen E Hilbrands LB Joosten I Rapamycin, and not cyclosporin A, preserves the highly suppressive CD27+ subset of human CD4 + CD25+ regulatory T cells Blood 2006 107 1018 1023 16210336 10.1182/blood-2005-07-3032

[B118] Jung J Choe J Li L Choi YS Regulation of CD27 expression in the course of germinal center B cell differentiation: the pivotal role of IL-10 Eur J Immunol 2000 30 2437 2443 10.1002/1521-4141(2000)30:8<2437::AID-IMMU2437>3.0.CO;2-M 10940936

[B119] Oshima H Nakano H Nohara C Kobata T Nakajima A Jenkins NA Gilbert DJ Copeland NG Muto T Yagita H Okumura K Characterization of murine CD70 by molecular cloning and mAb Int Immunol 1998 10 517 526 10.1093/intimm/10.4.517 9620608

[B120] Peperzak V Xiao Y Veraar EA Borst J CD27 sustains survival of CTLs in virus-infected nonlymphoid tissue in mice by inducing autocrine IL-2 production J Clin Invest 2010 120 168 178 10.1172/JCI40178 19955658 PMC2798690

[B121] Song DG Ye Q Poussin M Harms GM Figini M Powell DJ CD27 costimulation augments the survival and antitumor activity of redirected human T cells *in vivo* Blood 2012 119 696 706 10.1182/blood-2011-03-344275 22117050

[B122] Kelly JM Darcy PK Markby JL Godfrey DI Takeda K Yagita H Smyth MJ Induction of tumor-specific T cell memory by NK cell-mediated tumor rejection Nat Immunol 2002 3 83 90 11743585 10.1038/ni746

[B123] Claus C Riether C Schürch C Matter MS Hilmenyuk T Ochsenbein AF CD27 signaling increases the frequency of regulatory T cells and promotes tumor growth Cancer Res 2012 72 3664 3676 10.1158/0008-5472.CAN-11-2791 22628427

[B124] Schürch C Riether C Matter MS Tzankov A Ochsenbein AF CD27 signaling on chronic myelogenous leukemia stem cells activates Wnt target genes and promotes disease progression J Clin Invest 2012 122 624 638 10.1172/JCI45977 22232214 PMC3266773

[B125] Celldex Therapeutics initiates CDX-1127 phase 1 study in malignant solid tumors [press release] http://www.news-medical.net/news/20111108/Celldex-Therapeutics-initiates-CDX-1127-Phase-1-study-in-malignant-solid-tumors.aspx

[B126] He L-Z Thomas L Weidlick J Vitale L O’Neill T Prostak N Sundarapandiyan K Marsh H Yellin M Davis TA Keler T Development of a human anti-CD27 antibody with efficacy in lymphoma and leukemia models by two distinct mechanisms [Abstract] Blood 2011 118 abstr 2861

[B127] Vanneman M Dranoff G Combining immunotherapy and targeted therapies in cancer treatment Nature Rev Cancer 2012 12 237 251 10.1038/nrc3237 22437869 PMC3967236

[B128] Suntharalingam G Perry MR Ward S Brett SJ Castello-Cortes A Brunner MD Panoskaltsis N Cytokine storm in a phase 1 trial of the anti-CD28 monoclonal antibody TGN1412 N Engl J Med 2006 355 1018 1028 10.1056/NEJMoa063842 16908486

